# Association Between Fatigue and Motor Exertion in Patients With Multiple Sclerosis—a Prospective Study

**DOI:** 10.3389/fneur.2020.00208

**Published:** 2020-04-15

**Authors:** Daniel Drebinger, Ludwig Rasche, Daniel Kroneberg, Patrik Althoff, Judith Bellmann-Strobl, Martin Weygandt, Friedemann Paul, Alexander U. Brandt, Tanja Schmitz-Hübsch

**Affiliations:** ^1^NeuroCure Clinical Research Center, Charité—Universitätsmedizin Berlin, corporate member of Freie Universität Berlin, Berlin Institute of Health, Humboldt-Universität zu Berlin, Berlin, Germany; ^2^Department of Neurology, Park Clinic Weissensee, Berlin, Germany; ^3^Department of Neurology and Experimental Neurology, Charité—Universitätsmedizin Berlin, corporate member of Freie Universität Berlin, Humboldt-Universität zu Berlin, and Berlin Institute of Health, Berlin, Germany; ^4^Experimental and Clinical Research Center, Max Delbrueck Center for Molecular Medicine and Charité—Universitätsmedizin Berlin, corporate member of Freie Universität Berlin, Berlin Institute of Health, Humboldt-Universität zu Berlin, Berlin, Germany; ^5^Department of Neurology, University of California, Irvine, Irvine, CA, United States

**Keywords:** multiple sclerosis, fatigability, state/trait-fatigue, walking, balance

## Abstract

**Background:** Fatigue in multiple sclerosis (MS) is conceived as a multidimensional construct.

**Objectives:** This study aims to describe the changes of balance and gait parameters after 6 min of walking (6 MW) as potential quantitative markers for perceptions of state fatigue and trait fatigue in MS.

**Methods:** A total of 19 patients with MS (17 with fatigue) and 24 healthy subjects underwent static posturography, gait analysis, and ratings of perceived exertion before and after 6 MW.

**Results:** 6 MW was perceived as exhaustive, but both groups featured more dynamic comfortable speed walking after 6 MW. Shorter stride length at maximum speed and increased postural sway after 6 MW indicated fatigability of balance and gait in MS group only. While most changes were related to higher levels of perceived exertion after 6 MW (state fatigue), higher fatigue ratings (trait fatigue) were only associated with less increase in arm swing at comfortable speed. Further analysis revealed different associations of trait fatigue and performance fatigability with disability and motor functions. Performance fatigability was most closely related to the Expanded Disability Status Scale, while for trait fatigue, the strongest correlations were seen with balance function and handgrip strength.

**Conclusions:** Fatigability of performance was closely related to perceptions of exertion after 6 MW (state fatigue) and disability in MS but distinct from fatigue ratings, conceived as trait fatigue. Our study identified postural sway, arm swing during gait, and hand grip strength as unexpected potential motor indicators of fatigue ratings in MS.

## Introduction

Fatigue is a frequent and burdensome complaint even in the early disease stages of multiple sclerosis (MS) (1, 2), and there is a need for more effective treatment options (3). Although fatigue has been related to disability status, depressive symptoms, and brain connectivity in MS, there is no consensus on its etiology and the definitions of the constructs are still evolving (4, 5).

The term “fatigue” is used in different ways, ranging from unspecific (6) to more specific definitions, e.g., by the MS research council as “a subjective lack of physical and/or mental energy that is perceived by the individual or caregiver to interfere with usual and desired activities” (7). It is generally held that a sensation of fatigue does occur physiologically following an effort-demanding activity, while in the context of disease, fatigue may present as “pathological exhaustion” that occurs earlier, with lighter activity and more persistence or even independent of effort demands (8). Kluger et al. (9) proposed “perception of fatigue” and “performance fatigability” as discernible but related components of fatigue which both can present in the motor or the cognitive domain.

At the perceptional level, transient sensations of weariness or lack of energy during or right after exercise (perception of exertion) can be conceived as “state fatigue” (10), while pathological fatigue refers to a frequent, prolonged, or constant sensation over longer time frames which has been conceptualized as “trait fatigue” (11) and represents the construct assessed by the fatigue self-rating scales commonly used in MS (12, 13).

Performance fatigability as such is a physiological phenomenon. It can be described as a decline in performance with sustained activity and may be objectively quantified as the change of an appropriate performance parameter with prolonged exercise. Various performance measures and exertion paradigms have been explored for this purpose, but recent reviews concluded that there is currently no gold standard to assess fatigability in MS (14). As per one hypothesis, it has been suggested that fatigue as a symptom in MS may arise of altered interactions between perceptions of fatigue and fatigability and the relations of both to limitations in patients' functions, specifically mobility, need further study (11).

Table 1 sums up the fatigue taxonomy as used throughout this manuscript.

**Table 1:** Overview of fatigue taxonomy as used in this manuscript according to Kluger et al. (9), Wolff et al. (10), and Enoka and Duchateau (11).

**Construct**	**Definition**	**Assessment**
Perception of exertion (state fatigue)	Refers to the perception of fatigue in situations of effort-demanding activities that is physiologically transient and recovers with rest, understood as state fatigue	Here: self-rating (BORG) after 6MW standard exertion task alternatives: VAS ratings of state fatigue
Perception of (trait) fatigue	Refers to “pathological fatigue” as a frequent, prolonged, or constant disabling sensation of weariness and exhaustion over longer time frames, interfering with usual/desired activities, understood as fatigue trait	Here: self-rating (FSMC) alternatives: other fatigue self-rating scales (MFIS, FSS)
Performance fatigability	Refers to a reduced capacity to maintain activity which can be observed as a decline in performance measures with effort-demanding activities	Here: the delta-measure of balance and gait parameters after 6 MW standard exertion task Alternatives: other fatiguing paradigms (static and dynamic force production, treadmill) (6MW distance itself may reflect fatigability to some extent)

Given the high relevance of fatigue in MS and the ongoing efforts to find effective interventions (15), we aimed to evaluate the suitability of quantitative motor markers as measures of different components of MS fatigue. Specifically, in a first step, we described the extent of fatigability or the changes in motor parameters induced by participation in a 6-min walk test for groups of PwMS and healthy subjects. In a second step, the motor parameters indicating significant exertion effects across all participants or parameters varying between both groups with the regard to the exertion induced were related to perceived state fatigue and trait fatigue in PwMS. Additionally, these factors were also related to the patients' disability and specific motor functions.

## Methods

### Study Population

This prospective observational study was conducted at a university MS referral center. We included people with MS (PwMS), according to the 2017 revised criteria (16), who felt able to walk independently for 6 min (6 MW), including the use of unilateral walking aids, and healthy subjects (HC) of comparable age, gender ratio, and height (Table 2).

**Table 2:** Demographics, clinical data, and motor measures are described by group.

**Metric**	**PwMS**		**HC**		**Group difference**	
	**Mean (SD)**		**Mean (SD)**		***p*-value**	**Test**
Group size	19		24			
Age (years)	50.5	(9.5)	47.1	(17.1)	0.5	*t*-test
Height (cm)	175.6	(8.7)	172.4	(10.0)	0.3	*t*-test
Weight (kg)	78.4	(15.1)	70.4	(10.7)	0.047	*t*-test
BMI (kg/m^2^)	25.5	(5.1)	23.6	(2.6)	0.13	*t*-test
F:M	08:11		12:12		0.6	Chi-square
FSMC total	63.6	(19.0)				
FSMC cognitive	30.5	(10.3)				
FSMC motor	33.1	(9.4)				
PHQ-9^a^^,^^b^	7.3	(4.0)	2.1	(1.4)	<0.001	Mann–Whitney *U* test
EDSS	Median 3 (range: 1–6)					
6MW distance (m)	490.1	(134.6)	657.2	(87.4)	<0.001	*t*-test
VPC closed stance EO pre^c^ (°/s)	0.4	(0.2)	0.2	(0.1)	<0.001	*t*-test
VPC closed stance EC pre^d^ (°/s)	0.7	(0.3)	0.3	(0.1)	<0.001	*t*-test
Hand grip force dominant (kg)	40.2	(15.2)	42.3	(13.5)	0.6	*t*-test
Hand grip force non-dominant (kg)	33.7	(14.8)	40.8	(13.2)	0.1	Mann–Whitney *U* test
Borg pre	10.7	(2.9)	7	(1.5)	<0.001	Mann–Whitney *U* test
Borg post	14.0	(3.4)	10.1	(2.0)	<0.001	*t*-test
Δborg	3.3	(2.4)	3.1	(2.3)	0.9	*t*-test

*F, female; M, male; EDSS, Expanded Disability Status Scale; VPC, visual perceptive computing; EC, eyes closed; FSMC, Fatigue Scale for Motor and Cognitive Function; 6MW, 6-min walk; Borg, Borg Rating of Perceived Exertion; PHQ-9, Patient Health Questionnaire 9-item*.

a *Two PwMS were classified as major depressive syndrome according to PHQ-9 results (FSMC total: 47 and 80; Borg post 17 and 18) and for two other depressive syndromes (FSMC total: 73 and 88; Borg post 13 and 17)*.

b *Missing data from three healthy subjects*.

c *16 PwMS included; three PwMS were excluded because they were unable to stand on spot in closed stance with eyes open*.

d *14 PwMS included; five PwMS were excluded because they were unable to stand on spot in closed stance with their eyes closed*.

*Data are given as mean (SD) unless otherwise stated*.

Exclusion criteria were relapse within the last 30 days, comorbid neurological diagnosis, or any other condition with potential impact on movement functions.

### Ethics Statement

The study was approved by the local institutional review board (EA1/339/16, amendment 1) and conducted in accordance with the Declaration of Helsinki in its currently applicable version. All the participants provided written informed consent.

### Assessments

Severity of fatigue was assessed with the 20-item Fatigue Scale for Motor and Cognitive Functions questionnaire (FSMC) (13) classified as no fatigue (<43), moderate fatigue (43–62), and most severe fatigue (>62). The sub-scores of the FSMC motor and cognitive domain were reported in the tables, but statistical tests only applied to FSMC total, as recommended previously (17).

We screened for clinically relevant depressive syndrome by using a self-reported patient health questionnaire 9 item (PHQ-9), using algorithmic classification into none, other, or major depressive syndrome (18).

Disability in PwMS was rated with the expanded disability status scale (EDSS). We recorded three indicators of motor functions:

- postural sway in static posturography (closed stance with the eyes closed) as a relevant measure of balance function (19) recorded by visual perceptive computing using Microsoft Kinect™ and a custom-written software (version 2.0.1, Motognosis GmbH, Germany) (20),
- 6 MW distance as indicator of walking endurance with known validity against habitual walking performance in MS (21), and
- hand grip strength (dominant and non-dominant hand) as a non-locomotor indicator of general functional status and physical health (22), reported as the mean of three trials for each hand according to the Southampton protocol (23) (Jamar dynamometer; Patterson Medical, USA).

### Motor Fatiguing Paradigm

**Figure 1:**
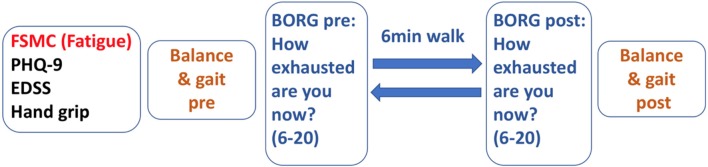
Time sequence of assessments in the 6 MW motor fatiguing paradigm.

Figure 1 depicts the test sequence of our paradigm. After general clinical and motor assessment, 6 MW was used as a moderate-intensity exercise expected to induce performance fatigability.

To verify the level of exertion induced by 6 MW, we documented the participants' ratings of perceived exertion [BORG, score range 6 (low) to 20 (high exertion)] (24) before 6 MW and again directly after 6 MW.

To quantify the fatigability of motor performance, the participants had balance and gait assessments before and immediately after 6 MW. Fatigability was then described as difference to the baseline. Posturography was assessed in closed stance with the eyes open, then followed with the eyes closed for 20 s each as described above. Gait was recorded with Mobility Lab™ (APDM Inc., USA) from 2 × 15-m level walks with *U*-turn around a cone. The subjects were instructed to walk at their comfortable speed and at their maximum walking speed two times each.

### Data Management and Pre-processing

One single item missing in FSMC was replaced by the mean of the remaining items of that subject. Missing items in motor parameters were considered per item and are reported with results.

Two patients performed 6 MW using a cane and one of them stopped performing the 6 MW early (4 min 25 s). These data points were included in the analysis but are indicated in the respective figures.

Data for posturography were missing for three PwMS who were unable to stand in closed stance. Two other PwMS were unable to perform closed stance with their eyes closed. Data from gait recordings were not available in one patient, and the arm and trunk parameters were missing in another four PwMS due to recording errors.

Sway during posturography was described as three-dimensional angular velocity of a vector movement of hip level mid-point relative to feet midpoint (20).

Gait parameters were calculated by the manufacturer's (APDM Inc., USA) algorithm for the plugin “Iwalk” (version 2.0) which provides algorithmic exclusion of the first steps of each walk and algorithmic definition of steps in turn. All trials were inspected for correct excision of turns (25). We additionally excluded the last stride of each trial and used only recordings with a minimum of six gait cycles. The mean of two repetitions was used for analysis. Table 3 gives an overview of all the motor parameters acquired in the fatiguing paradigm.

**Table 3:** Overview of balance and gait parameters recorded before and after 6-min walk.

**Method**	**Condition**	**Parameter**	**Abbreviation**	**Unit**
Visual perceptive computing	Stance	Sway closed stance eyes open angular speed 3D	EO 3D speed	Degrees/s
		Sway closed stance eyes closed angular speed 3D	EC 3D speed	Degrees/s
		Romberg ratio angular speed 3D	Romberg 3D	–
Mobility lab	Comfortable speed walking/maximum speed walking	Cadence	Cad	Steps/min
		Stride time	ST	s
		Gait speed	Speed	m/s
		Stride length	SL	m
		Circumduction	Circum	cm
		Arm swing velocity	Arm speed	Degrees/s
		Arm range of motion	Arm ROM	Degrees
		Coronal range of motion	Trunk roll ROM	Degrees
		Sagittal range of motion	Trunk pitch ROM	Degrees

### Statistical Analysis

The descriptive analyses included a calculation of group means and standard deviations (SD) (median/interquartile range for EDSS). Normality testing was performed for all parameters using the Shapiro–Wilk test and the statistical tests were chosen accordingly (see Table 2). All gait and balance parameters (see Table 3) were normally distributed by test and inspection of plots.

Between-group comparisons for demographic and clinical characteristics used *t*-test, Mann–Whitney *U* test, or chi-square test as indicated (Table 2).

The amount of change induced by 6 MW was described as raw difference (measure-post– measure-pre = delta-measure). A positive delta thus denoted a numerical increase after exertion. Measures pre-post were compared by *t*-test.

We applied ANOVA for repeated measures (type III) for each motor parameter with test repetition after 6 MW (effect of exertion), status of PwMS or HC (effect of group), and interaction (effect of exertion × group) as factors.

For motor parameters with significant exertion or interaction effect, we applied Spearman correlations within the PwMS group between fatigability (delta-measure) and perceptional ratings of state (BORG post) and trait (FSMC) fatigue.

In another step of the analyses, we used correlation analysis to explore the hypothesized triangular relation between (1) perceptions of state/trait fatigue, (2) performance fatigability, and (3) MS disability and limitations in motor functions (walking function, balance, and hand grip strength).

Significance was set at *p* < 0.05, but trends were reported for further analysis. The statistical analyses were performed with R version 3.5.1.

## Results

### Sample Characteristics

FSMC indicated relevant trait fatigue in all but two of the 19 PwMS included, with more than half classified as severe fatigue. Depression screening indicated relevant depressive symptoms in four subjects. PwMS were impaired in balance and walking function, while their hand grip strength did not clearly differ from HC (Table 2).

### Validity of Fatiguing Paradigm

**Figure 2:**
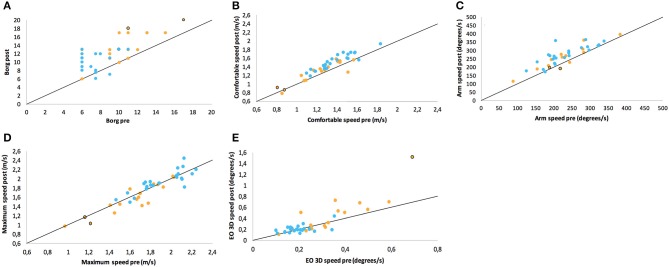
Scatter plots of ratings/performance before (pre) and after (post) exertion by 6 MW for **(A)** Borg ratings of perceived exertion, **(B)** comfortable gait speed, **(C)** arm swing in comfortable speed walking, **(D)** maximum gait speed, and **(E)** sway in static posturography in closed stance with the eyes open. Points on the line would represent the identical pre- and post-ratings (no change), while values above this line denote the numerical increase of the respective parameter. The groups are color-coded as PwMS (orange) and HC (blue). Two patients who walked with a cane are highlighted with a black curl.

The 6 MW, despite different individual distances walked, induced a perception of exertion in all participants, confirming validity as a motor fatiguing paradigm. We observed a three-point increase in BORG ratings after 6 MW in both groups (*p* < 0.001 PwMS, *p* < 0.001 HC; Table 2, Figure 2A), although perceived exertion in PwMS was generally higher than in HC even before 6 MW performance (Table 4).

**Table 4:** Differences within-group for people with multiple sclerosis (PwMS) and healthy subjects (HC) between balance and gait parameters taken before (pre) and after (post) exertion by 6-min walk.

**Test condition**	**Parameter**	**MS**			**HC**		
		**Pre mean (SD)**	**Post mean (SD)**	**Delta mean (SD)**	**Pre mean (SD)**	**Post mean (SD)**	**Delta mean (SD)**
BORG	Perceived exertion	10.500 (2.94)	13.889 (3.50)	3.389 (2.43)	7.000 (1.46)	10.146 (2.00)	3.146 (2.28)
Stance	EO 3D speed^a^	0.350 (0.15)	0.470 (0.34)	0.120 (0.23)	0.198 (0.06)	0.201 (0.07)	0.003 (0.06)
	EC 3D speed^b^	0.655 (0.33)	0.725 (0.42)	0.031 (0.28)	0.292 (0.11)	0.312 (0.11)	0.020 (0.09)
	Romberg 3D^b^	1.970 (0.59)	1.812 (0.74)	−0.154 (0.91)	1.508 (0.45)	1.595 (0.48)	0.087 (0.53)
Comfortable speed walking	Cad	110.412 (11.39)	112.335 (12.91)	1.924 (4.19)	117.912 (7.18)	122.766 (7.90)	4.854 (3.29)
	ST	1.099 (0.12)	1.084 (0.15)	−0.015 (0.05)	1.022 (0.06)	0.982 (0.06)	−0.040 (0.03)
	Speed	1.231 (0.23)	1.278 (0.25)	0.046 (0.10)	1.381 (0.18)	1.511 (0.19)	0.130 (0.07)
	SL	1.330 (0.17)	1.355 (0.17)	0.025 (0.06)	1.403 (0.16)	1.474 (0.15)	0.071 (0.05)
	Arm speed^c^	226.657 (69.25)	252.867 (73.32)	26.210 (27.65)	229.928 (57.53)	271.215 (55.60)	41.287 (35.34)
	Arm ROM^c^	53.418 (20.77)	60.486 (22.70)	7.068 (7.10)	58.404 (17.02)	66.631 (15.28)	8.227 (8.15)
	Trunk roll ROM^c^	7.446 (3.18)	8.794 (3.02)	1.348 (0.81)	6.039 (2.25)	7.626 (2.67)	1.587 (1.26)
	Trunk pitch ROM^c^	6.019 (2.52)	6.429 (2.47)	0.410 (0.92)	4.742 (0.97)	5.065 (1.06)	0.323 (0.76)
Maximum speed walking	Cad	127.138 (12.40)	125.418 (14.33)	−1.721 (5.41)	141.032 (8.58)	141.835 (8.50)	0.803 (5.76)
	ST	0.954 (0.10)	0.970 (0.12)	0.016 (0.04)	0.854 (0.05)	0.850 (0.05)	−0.005 (0.04)
	Speed	1.630 (0.29)	1.568 (0.32)	−0.062 (0.13)	1.911 (0.22)	1.928 (0.23)	0.016 (0.13)
	SL	1.529 (0.19)	1.487 (0.19)	−0.042 (0.07)	1.628 (0.18)	1.630 (0.18)	0.003 (0.05)
	Arm speed^c^	295.209 (65.62)	309.927 (73.46)	14.718 (31.54)	345.554 (81.65)	373.136 (97.58)	27.582 (50.66)
	Arm ROM^c^	66.994 (20.90)	67.579 (21.72)	0.585 (6.61)	78.209 (19.65)	83.133 (21.11)	4.925 (8.00)
	Trunk roll ROM^c^	8.412 (3.45)	9.224 (2.90)	0.812 (1.26)	7.388 (2.78)	8.509 (2.95)	1.120 (1.23)
	Trunk pitch ROM^c^	7.187 (2.86)	6.921 (2.78)	−0.266 (0.93)	6.076 (1.51)	6.595 (2.29)	0.519 (1.62)

*SD, standard deviation*.

*For all mobility lab parameters, one PwMS was excluded because of missing data*.

a *16 PwMS included; three PwMS were excluded because they were unable to stand on spot in closed stance with eyes open*.

b *14 PwMS included; five PwMS were excluded because they were unable to stand on spot in closed stance with their eyes closed*.

c *14 PwMS included; five PwMS were excluded because of missing data due to technical problems*.

*The disability and impairment scores were of similar size for these smaller sample sizes*.

### Motor Performance After Exertion

The balance and gait parameters recorded before and after 6 MW revealed a complex pattern of changes (Table 4). Worsening of balance, i.e., increase of postural sway (eyes open), was observed in PwMS only, not in HC (Figure 2E).

Concerning gait, a decline of performance in PwMS was only observed at maximum speed walking. Stride length decreased in PwMS only but remained stable in HC (Figure 2D). However, arm swing and trunk roll movements indicated larger ranges of upper body movement after 6 MW in both groups.

At comfortable speed walking, our observations clearly contrasted the expected decline of motor performance with exertion. The observed changes instead indicated a more dynamic walking pattern after 6 MW in both groups, although less pronounced in PwMS. Specifically, 6 MW induced an increase of comfortable gait speed and related parameters (cadence, stride time, and stride length) as well as increased arm and trunk motion. ANOVA revealed an interaction of the effect of exertion and group for several parameters (Table 5). Furthermore, an inspection of the respective plots suggested that the amount of change induced by 6 MW was dependent on the baseline value (Figures 2B–E). For example, Figure 2C indicates that lower arm swing velocity at baseline—occurring in PwMS with higher disability grades as well as in two elderly HC—coincides with less increase after 6 MW.

**Table 5:** Results of ANOVA with each gait and balance parameter as dependent variable and effect of exertion, group, and interaction as factors.

**Test condition**	**Parameter**	**Effect of exertion**		**Group**		**Interaction**	
		***p***	**Partial eta^**2**^**	***p***	**Partial eta^**2**^**	***p***	**Partial eta^**2**^**
Stance	**EO 3D speed^a^**	0.016	0.143	<0.001	0.311	0.022	0.130
	EC 3D speed^b^	0.426	0.018	<0.001	0.410	0.858	0.001
	Romberg 3D^b^	0.775	0.002	0.025	0.132	0.303	0.029
Comfortable speed walking	**Cad**	<0.001	0.463	0.005	0.183	0.015	0.139
	**ST**	<0.001	0.358	0.005	0.178	0.038	0.103
	**Speed**	<0.001	0.521	0.005	0.181	0.003	0.196
	**SL**	<0.001	0.465	0.062	0.085	0.007	0.166
	**Arm speed^c^**	<0.001	0.510	0.596	0.008	0.180	0.049
	**Arm ROM^c^**	<0.001	0.486	0.363	0.023	0.661	0.005
	**Trunk roll ROM^c^**	<0.001	0.629	0.158	0.055	0.529	0.011
	**Trunk pitch ROM^c^**	0.012	0.163	0.023	0.135	0.755	0.003
Maximum speed walking	Cad	0.603	0.007	<0.001	0.348	0.157	0.049
	ST	0.338	0.023	<0.001	0.337	0.090	0.070
	Speed	0.262	0.031	<0.001	0.286	0.059	0.086
	**SL**	0.041	0.100	0.040	0.101	0.022	0.125
	**Arm speed^c^**	0.008	0.180	0.042	0.110	0.398	0.020
	**Arm ROM^c^**	0.036	0.116	0.059	0.096	0.095	0.075
	**Trunk roll ROM^c^**	0.000	0.373	0.382	0.021	0.466	0.015
	Trunk pitch ROM^c^	0.596	0.008	0.335	0.026	0.106	0.071

For all mobility lab parameters, one PwMS was excluded because of missing data

a *16 PwMS included; three PwMS were excluded because they were unable to stand on spot in closed stance with eyes open*.

b *14 PwMS included; five PwMS were excluded because they were unable to stand on spot in closed stance with their eyes closed*.

c *14 PwMS included; five PwMS were excluded because of missing data due to technical problems*.

*P < 0.05 were deemed significant*.

*Parameters indicated in bold were selected for correlation analysis based on ANOVA results*.

### Relation of Performance Fatigability to Trait Fatigue and State Fatigue in Multiple Sclerosis

Next, we analyzed whether performance fatigability, i.e., delta induced by 6 MW, was associated with perception of state fatigue (BORG) or trait fatigue (FSMC) in MS (Table 6). Concerning state fatigue, those with higher ratings of perceived exertion after 6 MW featured less increase in speed, stride length, and arm movements at comfortable speed walking.

**Table 6:** Spearman correlations within PwMS group between changes in balance and gait parameters after 6 MW (delta parameter) to perception of fatigue (trait), perception of exertion, disability, and motor functions reported as rho (ρ).

		**Parameter**	**Perception of fatigue**			**Perceived exertion**	**Disability**	**Motor function**			
			**FSMC total**	**FSMC motor**	**FSMC cognitive**	**Borg post**	**EDSS**	**Gait**	**Balance**	**Hand grip strength**	
								**6-MW distance**	**Stance eyes closed pre^a^**	**Non-dominant**	**Dominant**
Perception of fatigue		FSMC total	1 (<0.001)	0.95 (<0.001)	0.95 (<0.001)	0.4 (0.090)	ns	ns	0.68 (0.010)	−0.57 (0.010)	−0.5 (0.029)
		FSMC motor		1 (<0.001)	0.84 (<0.001)	0.42 (0.070)	0.4 (0.089)	−0.42 (0.071)	0.71 (0.004)	−0.51 (0.027)	−0.48 (0.040)
		FSMC cognitive			1 (<0.001)	ns	ns	ns	0.6 (0.026)	−0.54 (0.017)	−0.47 (0.045)
Perceived exertion		Borg post				1 (<0.001)	0.77 (<0.001)	−0.71 (0.001)	0.59 (0.03)	ns	ns
Disability		EDSS					1 (<0.001)	−0.88 (<0.001)	0.67 (0.009)	ns	ns
Motor function	Gait	6–MW distance						1 (<0.001)	−0.76 (0.002)	0.4 (0.089)	ns
	Balance	Stance eyes closed pre							1 (<0.001)	ns	ns
	Hand grip strength	Non–dominant								1 (<0.001)	0.65 (0.003)
		Dominant									1 (<0.001)
Stance		ΔEO 3D speed^b^	ns	ns	ns	ns	ns	ns	0.61 (0.024)	ns	ns
Comfortable speed walking		ΔCad	ns	ns	ns	−0.46 (0.060)	ns	ns	ns	ns	ns
		Δ ST	ns	ns	ns	0.44 (0.070)	ns	ns	ns	ns	ns
		Δ Speed	ns	ns	ns	−0.51 (0.030)	−0.48 (0.044)	0.43 (0.077)	ns	ns	ns
		Δ SL	ns	ns	ns	−0.49 (0.040)	−0.45 (0.060)	ns	ns	ns	ns
		Δ Arm speed^c^	−0.7 (0.007)	−0.47 (0.087)	−0.76 (0.002)	−0.55 (0.040)	−0.64 (0.013)	0.61 (0.022)	ns	0.62 (0.020)	ns
		Δ Arm ROM^c^	−0.83 (<0.001)	−0.72 (0.004)	−0.78 (0.001)	−0.62 (0.020)	−0.73 (0.003)	0.77 (0.002)	−0.72 (0.019)	0.56 (0.040)	ns
		Δ Trunk roll ROM^c^	−0.52 (0.062)	ns	−0.53 (0.050)	ns	ns	ns	ns	ns	ns
		Δ Trunk pitch ROM^c^	ns	ns	ns	ns	ns	ns	ns	ns	ns
Maximum speed walking		Δ SL	ns	ns	ns	ns	ns	ns	ns	ns	ns
		Δ Arm speed^c^	ns	0.51 (0.062)	ns	ns	ns	ns	ns	ns	ns
		Δ Arm ROM^c^	ns	ns	ns	ns	ns	ns	−0.56 (0.090)	ns	ns
		Δ Trunk roll ROM^c^	ns	ns	ns	ns	ns	ns	ns	ns	ns

a *14 PwMS included; five PwMS were excluded because they were unable to stand on spot in closed stance with their eyes closed*.

*For all mobility lab parameters, one PwMS was excluded because of missing data*.

b *16 PwMS included; three PwMS were excluded because they were unable to stand on spot in closed stance with eyes open*.

c *14 PwMS included; five PwMS were excluded because of missing data due to techincal problems*.

*For clarity, we only depict correlations with p < 0.1*.

Those with higher trait fatigue featured less increase of arm movements at comfortable speed walking, while no significant correlation was seen for the delta of any other balance or gait parameter. Furthermore, the subjects' ratings of state fatigue (BORG after 6 MW) were only weakly related to their ratings of trait fatigue (FSMC) (*r* = 0.396, *p* = 0.09).

### Relation of Performance Fatigability, State Fatigue, and Trait Fatigue to Disability or Limitations of Motor Functions in Multiple Sclerosis

Performance fatigability—specifically delta of arm range of motion at comfortable speed walking—and state fatigue after 6 MW, were very similarly related to EDSS (*r* = −0.73 and 0.77), walking endurance (*r* = 0.77 and −0.71), and balance function (*r* = −0.72 and 0.59). In addition, delta of arm movement was related to grip strength in the non-dominant hand and delta of postural sway (eyes open) after 6 MW was related to baseline balance function (*r* = 0.61).

**Figure 3:**
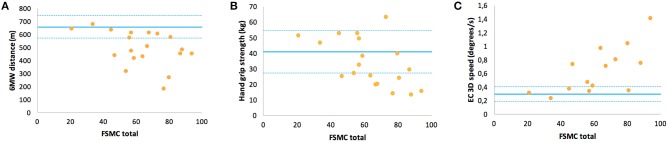
Correlations of the Fatigue Scale for Motor and Cognitive Functions questionnaire ratings of fatigue trait with specific motor function in multiple sclerosis: **(A)** walking function (6MW distance), **(B)** handgrip strength of the non-dominant hand, and **(C)** balance function (EC 3D speed). For interpretation, the means and the range of one standard deviation (solid and dashed lines) of HC are inserted.

In contrast, the FSMC ratings, i.e., levels of trait fatigue, were rather unrelated to EDSS (*r* = 0.34, *p* = 0.13) or walking endurance (*r* = −0.39, *p* = 0.1) but showed correlations to balance function (*r* = 0.68) and hand grip strength (*r* = −0.57 for non-dominant hand) (Table 6). An inspection of the respective data plots (Figures 3A–**C**) revealed that the subjects with motor functions outside the normal range consistently featured relevant trait fatigue but not *vice versa*.

## Discussion

We here report on changes of balance and gait functions after fatiguing exercise and their relation to perceptions of fatigue as well as disability and motor function in MS. In extension to many other studies, we aimed to separate fatigue as a trait variable conceived as the underlying construct of commonly used fatigue rating scales and as a state variable conceived as the actual perception of exertion induced by our 6 MW fatiguing paradigm. Our sample of PwMS featured high trait fatigue according to FSMC and low to moderate disability according to EDSS as reflected in limited walking endurance and balance function when contrasted to HC.

First, we were able to confirm that 6 MW is a fatiguing paradigm as perceptions of exertion increased after 6 MW. That the amount of change was similar in both groups despite different distances walked can be interpreted as an interaction of perceptions of (state) fatigue and fatigability, which leads to corresponding adjustments of performance (11).

Despite this, the changes observed for gait parameters when assessed before and immediately after 6 MW cannot be straightforwardly interpreted as expression of fatigability. The changes instead indicate a more dynamic gait pattern at comfortable speed walking after 6 MW in both groups with increased speed and larger arm swing and trunk movements. This observation was unexpected and, to our knowledge, this has not been described previously. This effect may combine a carry-over effect [which has been reported in other contexts (26)] from prior walking in fast pace for 6 min on the one hand and, on the other, an artifact of instructed short distance recording which may not reflect the walking speed an individual would usually assume for longer distances. In fact, 6 MW was shown to have higher validity against daily walking functions in MS compared to short-term recordings (27). The correlations of observed changes in comfortable speed walking with the level of state fatigue after 6 MW and 6 MW distance itself indicate that a “lower amount of increase” in gait parameters can be understood as expression of fatigability with delta of arm swing as the most relevant marker in this respect. Arm swing behavior is generally understudied (28) and we are not aware of its description in MS, which makes this marker difficult to interpret. As arm swing may influence recovery after tripping and improve gait efficiency and stability (28), further exploration of the arm swing behavior and the determinants in MS is highly relevant. Interestingly, in this study, arm swing also increased with exertion at maximum speed walking despite a decrease in stride length and gait speed observed in PwMS. The latter effect was not strong enough to consider it as a promising marker of performance fatigability in MS but is consistent with a decrease in walking speed reported from continuous or minute-by-minute 6 MW recordings (29, 30). As a limitation of our study, such recordings were not applied and thus respective deceleration indices could not be calculated for direct comparison. Taken together, the standardized short assessment battery of gait function applied in an appropriate fatiguing paradigm did not yield clinically applicable markers of fatigability.

The decline in balance function with exertion confirms previous reports (31). Future studies need to explore the relevance of this finding for fall risk and walking capabilities in MS. Worsening of balance with exertion has also been seen in elderly HC with prompt recovery after minutes (32) and has also been proposed for screening of workplace fatigue (33). The high susceptibility of balance function to effects of exertion surely warrants consideration in assessment protocols for balance testing. However, a more direct link of perceived fatigue to vestibular functions has been the topic of recent investigations (34), while the associations of balance function and psychological factors are largely unexplored. Of note is that the posturography system used in our study as a low-cost tool may be of potential utility for such research.

Performance fatigability, i.e., parameters' delta with exertion, was similarly correlated to BORG ratings after 6 MW and 6 MW distance, which both also had similar and robust correlations with disability and limitations of gait and balance function in MS. This is in line with previous evidence (30). It suggests that patient ratings of state fatigue after standard exercise as well as actual performance in the endurance task may both serve as good proxies for fatigability in MS. It further implies that (motor) fatigability in MS can be considered as a phenomenon closely related to the motor symptoms of the disease. The applicability of this finding in the cognitive domain or in other disease conditions needs further investigation.

As a general limitation, FSMC was not performed in HC and gender ratio between groups was not perfectly balanced. This may contribute to the underestimation of between-group difference due to a higher prevalence of fatigue in females at the population level. For FSMC correlations in PwMS, the sample size was small and included only two subjects without relevant fatigue (who were mainly unimpaired in all motor function tests). Nevertheless, in line with our results, the divergence of trait fatigue and disability in MS has been reported in larger cohorts with almost identical coefficients (12, 35) as well as the divergence of trait fatigue and fatigability (36). The occurrence of strong trait fatigue in the absence of other neurological symptoms or structural CNS change in chronic fatigue syndrome may be taken as another piece of evidence (37). Similar to our results, they (37) also found a relation of hand grip strength to the levels of trait fatigue, including a sample of PwMS. This further supports divergence between trait fatigue and MS-related disability as handgrip strength was unrelated to EDSS/6 MW and did not clearly differ from HC in our study. There is ample evidence for handgrip strength as an indicator of general health status with relations to morbidity and mortality (22), and it is one of the five criteria to define frailty in the elderly (in combination with comfortable walking speed, weight loss, physical activity, and perceptions of exhaustion) (38). Future studies on hand grip strength in MS should aim to further explore and consider possible confounders (e.g., gender, age, and body weight) to gain more robust findings.

With this in mind, what is measured by fatigue rating scales as a disabling symptom in MS may be conceived as only indirectly or in a large part even non-related to MS disability as assessed by EDSS. In fact, previous studies determined depressive symptoms as the only independent predictor of FSS ratings (12). Four depressive cases on our study scored high on FSMC and Borg post, but their motor performance in the 6 MW paradigm seemed not distinct and *post hoc* analysis excluding these cases did not change the essence of the results. Other possible determinants such as general health, comorbidity (and subsequent medications), physical fitness, and pain remain largely unexplored to date. Further exploration of medication effects was not feasible in our study due to the small sample size and the various drugs applied (see Table 1 of the Supplementary Material). Mechanistic studies suggested self-control (10) or internal effort–reward trade-offs (39) as other possibly relevant psychological factors in MS fatigue. While a relation of trait fatigue to balance function has been reported previously (40), we were also able to link fatigability/state fatigue to balance function. Such interaction may be interpreted as worse balance being the expression of MS disability (with impact on fatigability) and also being influenced by other factors that at the same time impact on the levels of trait fatigue. In this respect, the impact of psychological determinants, e.g., attentional control, affective state, or general health, on balance function deserves further exploration. In sum, the correlation results are in line with the recently proposed distinction between the effort-dependent and the effort-independent components of MS fatigue (8) that may prove more useful as a concept than the performance–perception duality proposed by Kluger et al. (9).

Our study adds important aspects to inform future studies on MS fatigue. State levels of fatigue (e.g., Borg ratings) after standard exercise or 6 MW distance may serve as indicators of fatigability/effort-dependent component of MS fatigue that can be considered close to MS disability but distinct from patient ratings of (trait) fatigue. Handgrip strength and balance warrant further exploration, including the determination of appropriate testing and normalization procedures, as possibly useful indicators of trait fatigue. Easy means of assessment and ample normative data for both measures support their clinical applicability. Arm swing during gait may be an additional feature of interest with possible cross-correlations to other yet undetermined factors.

## Data Availability Statement

The dataset for this article is stored at Charité—Universitätsmedizin as patient consent did not include the publication of individual data.

## Ethics Statement

Ethical approval was obtained by the Ethics Committee of Charité—University Hospitals Berlin. The study was conducted in accordance to the Declaration of Helsinki in its currently applicable version and to the applicable German laws. Written informed consent was approved by all participants.

## Author Contributions

AB, DD, FP, JB-S, LR, MW, and TS-H contributed to the conception and design of the study. DD, DK, JB-S, LR, PA, and TS-H contributed to the acquisition and preprocessing of data. DD and TS-H performed the statistical analysis and drafted the first version of the manuscript. All authors reviewed the manuscript for intellectual content.

### Conflict of Interest

The authors declare that the research was conducted in the absence of any commercial or financial relationships that could be construed as a potential conflict of interest.
