# Supplementary material for: Association Between Fatigue and Motor Exertion in Patients With Multiple Sclerosis—a Prospective Study
Source: Front Neurol. 2020 Apr 15;11:208. doi: 10.3389/fneur.2020.00208 (PMC7174662; doi:10.3389/fneur.2020.00208)
Supplement: Supplementary file 1 [file Table_1.pdf]

Supplementary Material

Supplementary Table 1:
Full list of medication for PwMS

Patients	DMT	antidepressants	fampridine	other CNS	other non CNS
1	teriflunomide				
2					L-thyroxin
3	fingolimod		fampridine	baclofen	vitamin D
4					vitamin D
5	IFN beta				
6	IFN beta			levodopa	candesartan, HCT
7				gabapentin	amlodipine, metoprolole, ASS, pantoprazole, vitamin D
8	natalizumab	escitalopram			
9	fingolimod				L-thyroxin, ramipril, HCT, amlodipin
10	dimethylfumarat				propaverin
11		mirtazapin		pramipexol	allopurinol, lisinopril, HCT, bisoprolol, vitamin B12, folic acid
12	teriflunomide				vitamins D, B12, folic acid
13	fingolimod			gabapentin	L-thyroxin
14	dimethylfumarate				L-thyroxin
15	fingolimod	citalopram	fampridine	hydromorphon, ibuprofen, novaminsulfon	L-thyroxin, trospiumchlorid
16	natalizumab			cannabinoids	L-thyroxin, tamsulosin, vitamin D, darifenacin
17					L-thyroxin
18					
19				gabapentin	L-thyroxin, vitamin D

DMT: Disease modifying therapies; CNS: central nervous system; IFN beta: Interferon beta; HCT: Hydrochlorothiazide; ASS: Acetylsalicylic acid
